# Une complication exceptionnelle du tétanos généralisé: l’embolie pulmonaire

**DOI:** 10.11604/pamj.2016.25.172.9875

**Published:** 2016-11-17

**Authors:** Kais Regaieg, Mabrouk Bahloul, Rahma Gargouri, Abir Bouattour, Hèdi Chelly, Mounir Bouaziz

**Affiliations:** 1 Service de Réanimation Polyvalente, CHU Habib Bourguiba, Sfax, Tunisie

**Keywords:** Tétanos, embolie pulmonaire, physiopathologie, Tetanus, pulmonary embolism, physiopathology

## Abstract

Le tétanos est une maladie caractérisée par une paralysie spastique et des spasmes. C’est une pathologie grave. Elle nécessite une prise en charge en milieu de réanimation. La mortalité est essentiellement liée aux complications neurovégétatives et infectieuses. Les complications thromboemboliques sont exceptionnelles au cours de cette maladie. A notre connaissance, l’embolie pulmonaire n’a jamais été rapportée et confirmée au cours du tétanos généralisé. Nous présentons à travers cette observation un cas de tétanos généralisé compliqué d’une embolie pulmonaire fibrino cruorique.

## Abstract (en)

Tetanus is a disease characterized by spastic paralysis and spasms. It is a serious pathology that requires treatment in the ICU. Mortality rate is primarily due to neurodegenerative and infectious complications. Thromboembolic complications are rare. To the best of our knowledge, pulmonary embolism has never occorred and been confirmed in patients with generalized tetanus. Our study reports the case of a patient with generalized tetanus complicated by fibrin, cruoric pulmonary embolism.

## Introduction

Le tétanos est une maladie caractérisée par une paralysie spastique et des spasmes. Elle est due à une neurotoxine très puissante produite par le bacille anaérobie à Gram positif *clostridium tetani*. C’est une pathologie grave qui reste toujours d’actualité. Sa prise en charge se fait en milieu de réanimation. Plusieurs complications peuvent survenir au cours de l’évolution de cette maladie [1]. Les complications thromboemboliques sont exceptionnellement décrites. A notre connaissance, l’embolie pulmonaire n’a jamais été rapporté au cours du tétanos généralisé. Nous rapportons dans cette observation un cas de tétanos compliqué par une embolie pulmonaire et nous présentons des explications physiopathologiques de la rareté de cette complication et des hypothèses du mécanisme de sa survenue.

## Patient et observation

Il s’agit d’un homme âgé de 61 ans, pécheur, sans antécédents pathologiques notables, consulte aux urgences pour myalgie intense et trismus. L’histoire de la maladie remonte à deux semaines avant la consultation quand le patient s’est blessé au niveau de la plante du pied gauche avec un outil de jardinage souillé. Une désinfection locale a été faite et le patient n’a pas consulté un médecin. Son dernier vaccin anti tétanique datant depuis quarante ans au service militaire. L’examen initial aux urgences a montré un trismus, une diminution de l’ouverture palpébrale et une dysphagie. Ensuite, des contractures généralisées permanentes et douloureuses sont apparues avec accès d’opisthotonos. Devant ce tableau, le diagnostic de tétanos généralisé dans sa forme grave a été cliniquement posé.

Le patient a été transféré en réanimation. Il a été intubé ventilé sédaté, secondairement trachéotomisé. Il a reçu une injection intra musculaire de sérum anti tétanique (3000 UI) et une première injection de vaccin anti tétanique. Il a bénéficié d’une curarisation par du cisatracurium associé une benzodiazépine à base de Diazépam pour lutter contre les contractures musculaires et d’une analgésie opioïde par du fentanyl. Une antibiothérapie à base de métronidazol à dose 1.5 g/24h visant le *clostridium tétani* a été prescrite pour sept jours. Une anti coagulation préventive à base de l’enoxaparine à la posologie de 4 000 UI anti-Xa (0,4 ml) à raison d´une injection quotidienne en sous cutané a été administrée dès le premier jour, ainsi que des bas de contention ont été placés au niveau des deux membres inférieurs.

L’évolution a été marquée au cours de la première semaine par la persistance des contractures musculaires et des spasmes qui étaient réapparus à la moindre stimulation. Le contrôle de ces spasmes était difficilement obtenu par l’augmentation des doses et la prolongation de la durée de la curarisation. Par ailleurs, le patient était stable sur le plan hémodynamique avec une absence du syndrome dysautonomique, et sur le plan respiratoire avec des échanges gazométriques satisfaisantes.

Au huitième jour d’hospitalisation, le patient a présenté une hypoxémie non expliquée avec une dégradation du rapport gazométrique PaO2/FiO2 passant de 450 à 220. En fait, le patient était trachéotomisé ventilé et sédaté. Les curares sont arrêtés depuis 24 heures. Il était apyrétique. Sur le plan hémodynamique, il avait une fréquence cardiaque à 80 battements/min et une pression artérielle aux alentours de 130/70 mm Hg sans recours aux catécholamines. Sur le plan respiratoire, le patient était bien adapté au respirateur avec une auscultation strictement normale et des aspirations trachéales qui étaient claires. Les mollets étaient souples. La radiographie thoracique était normale.

**Figure 1:**
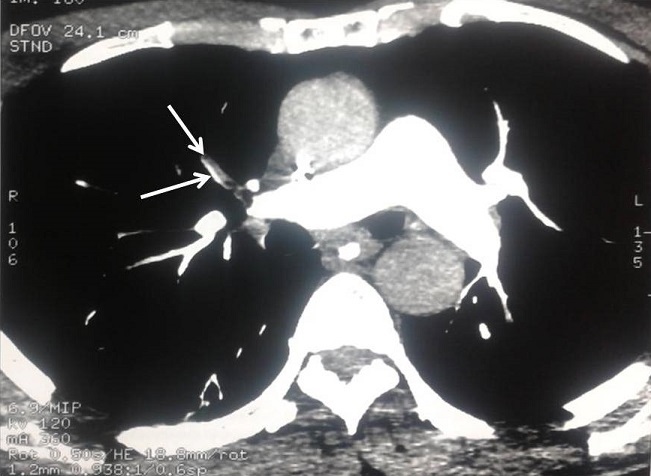
Défect d’opacification complet de l’artère segmentaire ventrale du lobe supérieur du poumon droit

L’électrocardiogramme ne montrait pas de modifications électriques. L’échographie des membres inférieurs était sans anomalie. Devant cette hypoxémie non expliquée, un angioscanner thoracique a été réalisé et a montré un défect d’opacification complet de l’artère segmentaire ventrale du lobe supérieur du poumon droit (Figure 1). Ainsi, le diagnostic d’embolie pulmonaire proximale a été confirmé et le patient a été mis sous une anti coagulation curative avec relais secondairement par les antivitamines K. Le bilan de thrombophilie (antithrombine III, protéine C et S, homocystéinémie, anticorps anti-noyaux et antiphospholipides, mutation du gène des facteurs II et V) ainsi que la recherche d’anticorps anti-cytoplasme des polynucléaires neutrophiles étaient négatifs chez ce patient. L’évolution ultérieure était favorable après un séjour de un mois en réanimation.

## Discussion

L’embolie pulmonaire est une complication classique des patients en réanimation. Cependant, les patients ayant un tétanos généralisé ne font pas généralement de complications thromboemboliques [1]. A notre connaissance, c’est le premier cas d’embolie pulmonaire qui a été rapporté chez un patient ayant un tétanos généralisé.

La physiopathologie de l’embolie pulmonaire est bien connue. En fait, la maladie commence souvent par un thrombus au niveau des membres inférieurs. Ce thrombus va ensuite migrer le long des veines fémorales, iliaques et la veine cave inférieure pour arriver dans le ventricule droit où il sera envoyé par la suite au niveau du tronc de l’artère pulmonaire ou dans ses branches de division entrainant des conséquences respiratoires et hémodynamique. Il existe trois facteurs principaux qui exposent à la formation de ce caillot, tel qu’il était décrit par Virchow : la lésion vasculaire, la stase sanguine et l’hypercoagulabilité [2]. Les patients en milieu de soins intensifs sont à haut risque de thrombose veineuse profonde et d’embolie pulmonaire [3]. Ce ci ne s’applique pas aux patients présentant un tétanos généralisé. Le tétanos est une maladie neurologique due à l’action de la toxine tétanique. La contamination d’une plaie par *clostridium tetani* qui est une bactérie de l’environnement, permet sous certaines conditions sa multiplication et la production locale de toxine. Celle-ci, est ensuite véhiculée dans la forme généralisée par les motoneurones jusqu’au système nerveux central par le transport rétrograde axonal et gagne, via les racines ventrales, les cornes ventrales de la moelle épinière ou les noyaux des nerfs crâniens moteurs qui contiennent les corps cellulaires des motoneurones. L’effet de la toxine tétanique s’exerce sur les interneurones inhibiteurs de la moelle épinière. De ce fait, le blocage de l’action régulatrice sur les motoneurones désorganise complètement les réflexes spinaux menant ainsi à une stimulation simultanée des muscles fléchisseurs et extenseurs. Les réflexes à des stimuli sensoriels ne sont plus inhibés par les commandes volontaires du cerveau et sont donc exacerbés. Les spasmes d’abord intermittents interviennent en réponse à des stimuli, ils deviennent ensuite de plus en plus fréquents puis permanents. Ils se traduisent par de violentes contractions douloureuses qui provoquent une posture en opisthotonos. Les membres sont rigidifiés et difficilement mobilisables [4]. Cette rigidité au niveau des membres inférieurs suite au spasme et à la contracture musculaire permet de lutter en permanence contre la stase sanguine. C’est une véritable pompe musculaire qui s’oppose également à l’effet de la gravité en interrompant périodiquement la colonne sanguine par écrasement périodique des veines profondes.

La survenue d’une embolie pulmonaire fibrino-cruorique chez notre patient peut être expliquée par plusieurs mécanismes. Tout d’abord, il s’agit d’un sujet âgé (âge supérieur à 60 ans) chez qui l’incidence d’embolie pulmonaire est très élevée. En effet, l’âge est un élément majeur pulmonaire avec une augmentation quasi exponentielle de la maladie thromboembolique après 40 ans [5]. Ce ci est expliqué par l’installation avec l’âge d’une anomalie de l’endothélium vasculaire avec une baisse de la sécrétion du monoxyde d’azote et des prostacyclines qui ont un rôle antiagrégant [3]. Ensuite, la prise en charge d’un patient atteint de tétanos généralisé nécessite le recours à une sédation profonde à base de morphiniques et de benzodiazépines ce qui favorise la survenue de complications thromboemboliques par augmentation de la durée de l’immobilisation et par la diminution des résistances vasculaires périphériques notamment une veinodilatation [6]. Ce ci est du à un effet inhibiteur sur le système nerveux sympathique par une dépression des barorécepteurs et une diminution de la sécrétion de la noradrénaline [7]. Enfin, il y avait un recours à une curarisation continue et prolongée. Les curares non dépolarisants, qui sont utilisés dans ces situations, sont des antagonistes de l’acétylcholine, induisant un bloc par compétition, et ainsi une myorelaxation qui s’oppose à la rigidité musculaire vue au cours du tétanos [8]. Dans notre observation, nous pensons que le patient a développé cette complication sous sédation et curare. Ainsi à l’arrêt des curares et la reprise du tonus, le thrombus a migré entrainant une embolie pulmonaire.

## Conclusion

Le tétanos généralisé est une pathologie redoutable encore largement présente dans certaines parties du monde. Elle nécessite une prise en charge dans un milieu de soins intensifs. La mortalité est due essentiellement aux complications neurovégétatives et infectieuses. Par contre, les complications thromboemboliques sont exceptionnelles au cours de cette maladie mais elles peuvent exister. La prévention reste un élément primordial que se soit pour lutter contre le tétanos, soit pour éviter les complications thromboemboliques au cours de la prise en charge de cette maladie.
